# Anxiety and depression in healthcare workers are associated with work stress and poor work ability

**DOI:** 10.3934/publichealth.2024063

**Published:** 2024-12-13

**Authors:** Nicola Magnavita, Igor Meraglia, Matteo Riccò

**Affiliations:** 1 Occupational Epidemiology and Health Unit, Department of Life Sciences and Public Health, Università Cattolica del Sacro Cuore, Roma, Italy; 2 Prevention and Safety Service in Workplaces (SPSAL), Local Sanitary Unit of Reggio Emilia, Reggio Emilia, Italy

**Keywords:** mental disorders, overcommitment, job satisfaction, mental health, workplace health promotion, effort/reward imbalance, disability management, COVID-19, quality of care, medical surveillance

## Abstract

**Background:**

Symptoms of anxiety and depression are very common among healthcare workers (HCWs) and could impact the quality of care.

**Objective:**

This study aimed to evaluate the prevalence of these disorders in a public health company and their association with work ability and work-related stress.

**Methods:**

A cross-sectional study involved 80 HCWs being treated for mental disorders (MD), 55 HCWs who said they suffered from MD but were not being treated, and 824 healthy colleagues. All workers completed the Work Ability Index (WAI), the Siegrist's Effort/Reward Imbalance questionnaire (ERI), the Goldberg's scales of anxiety and depression (GADS), and the Warr's scale of job satisfaction.

**Results:**

Three-quarters of workers with MD suffered from anxiety and/or depression. Workers who declared at the periodic medical examination in the workplace that they were being treated for MD had significantly lower levels of work ability than those of their colleagues who declared good mental health. They also reported greater work stress (high effort, low rewards, high overcommitment) and lower job satisfaction than their healthy colleagues. Symptomatic but untreated workers reported significantly lower work ability, lower satisfaction, and greater occupational stress than their healthy colleagues. In the entire sample, there were many workers with symptoms of anxiety or depression who did not declare these disorders during the examination. Overall, there were 328 suspected cases of anxiety (34.2%) and 334 cases of depression (34.8%). Anxious workers [*OR* = 8.11, 95% confidence interval (*CI*) = 3.74–17.58] and depressed workers (*OR* = 4.49, 95% *CI* = 2.22–9.10) had an increased risk of being classified as having “poor work ability”.

**Conclusion:**

The negative association between psychological symptoms and work ability even in undiagnosed/untreated workers demonstrates the usefulness of screening for these symptoms in work environments.

## 1. Introduction

Symptoms of anxiety and depression are very common among healthcare workers (HCWs). The recent COVID-19 pandemic highlighted the fact that many of them suffer from these disorders. The abundant scientific literature produced on this subject during the pandemic has been collected in a series of systematic reviews and meta-analyses [1]–[26]. The extent of the literature has drawn attention to how important the mental health of HCWs is, especially during health emergencies [27],[28], and has prompted the implementation of measures to control the risk of stress and burnout during epidemics [29]–[32].

However, this issue is much more widespread and not limited just to pandemics. HCWs are constantly exposed to numerous factors that can lead to anxiety and depression, which reduces their work ability. Mental disorders (MDs) are known to affect the health of HCWs and their ability to work, for example by increasing the risk of musculoskeletal disorders and absenteeism [33]. Workplace violence, to which HCWs are ubiquitously exposed, is associated with anxiety and depression [34] and poor mental health [35],[36]. Night work could also be a causative agent of anxiety and depression in HCWs [37],[38], as well as in other categories of workers [39]. Anxiety and depression in sleep-deprived HCWs have been associated with road accidents, near-misses, and medical errors [40]. Medical errors are inversely associated with compassion [41]. Compassion fatigue may be a major causative agent of impaired mental health for HCWs [42],[43]. The experience of fatigue by critical care nurses can increase the possibility of practice errors [44]. This creates a vicious circle because critical incidents in patient care are also associated with anxiety, depression, and post-traumatic stress disorder among staff [45]. Moreover, the state of mind experienced in case of medical errors involves HCWs from an emotional point of view [46]. Medical errors can often induce legal litigation, and malpractice stress is a well-known problem for HCWs [47]. HCWs' excessively sedentary lifestyle may also be associated with mental health problems. Longitudinal studies have shown that reduced physical activity in HCWs is associated with low mental health [48]. Taken together, many psychosocial factors act on HCWs and can determine excessive work-related stress associated with anxiety and depression [49]. Prolonged stress can induce burnout [50], a disorder that is associated with depression and anxiety [51] and that can have significant consequences for work organization and patients' safety and health [52]. For this reason, there have been numerous interventions to reduce the risk of stress and burnout in HCWs [53]–[55]. Prevention has been based on mindfulness-based stress reduction [56]–[58] or other interventions at the individual level to reduce occupational stress [59] and manage anxiety or depression [60],[61]. The attention given to this problem is due to the fact that HCWs' mental health is a critical issue because conditions that impair the healthcare worker's judgment can endanger the health of patients [62]–[65].

This brief overview is a good illustration of how the mental health of HCWs is constantly being challenged by exposure to numerous risk factors and is of critical importance to ensure the quality of healthcare. It is surprising that, although HCWs are exposed to many factors inducing anxiety or depression, few studies have investigated the effect that subclinical anxiety or depression, so frequent in HCWs, may have on their work ability.

Work ability concerns the capacity to manage job demands in relation to physical and psychological resources. This basic definition does not adequately describe the complexity of this concept, which has not been unambiguously established [66]. According to Ebener and Hasselhorn [67], it is the result of a multitude of items referring to health and functional capacities, knowledge, skills, values, attitudes, motivation, occupational situation, and demands. Tengland [68] observed that work ability includes a number of aspects such as health, basic standard competence, occupational competence, occupational qualities, and motivation, all related to the work tasks and the work environment. The commonest instrument for assessing work ability is the Work Ability Index (WAI). This tool, created in the 1980s by Finnish researchers, has been used in clinical and occupational epidemiology in many countries around the world. Work ability measured with the WAI is inversely proportional to the frequency of errors and cognitive failures in nurses [69] and with mental resources used to cope with job demands in physicians [70].

In order to shed light on this complex set of problems, we wanted to ascertain whether the presence of anxiety and depression in HCWs is associated with occupational stress and reduced work ability. We invited workers in a public health company to participate in an initiative to promote mental health that included screening for anxiety and depression and the assessment of occupational stress and work ability. The hypotheses we formulated were as follows:

1. Workers who report suffering from MD during their medical examination have worse work ability than others.
2. Anxiety and depression are present in the majority of those suffering from MD.
3. There may be cases of subclinical anxiety or depression even among workers who do not report suffering from MD.
4. Anxiety and depression are associated with occupational stress and poor job satisfaction.
5. Those who suffer from subclinical anxiety or depression have worse work ability than other workers.

Our intervention was therefore designed to evaluate the prevalence of subclinical anxiety and depression, to identify cases of possible pathology, and to bring them to the attention of a general practitioner or specialist. Furthermore, it aimed to provide the company with data on the spread of the phenomenon and its relevance for staff work ability to encourage the adoption of organizational and/or individual measures that could control it.

## 2. Materials and methods

### 2.1. Population and study design

The study was cross-sectional and was conducted from January 1 to December 31, 2019. In Italy, workers who are exposed to occupational risks are subjected to health surveillance that includes routine medical examinations in the workplace. In this study, we used data provided by workers from a public health company. According to good occupational health practices, workers who must undergo this mandatory examination are asked to declare any pathologies they suffer from or are being treated for. During the medical examination, the occupational doctor asked the worker: “Do you suffer from mental disorders such as depression, anxiety, insomnia, or burn-out?”. The doctor also investigated the presence of musculoskeletal diseases in the back, limbs, or other parts of the body, skin disease, cardiovascular disease (e.g., hypertension, coronary heart disease), endocrine or metabolic diseases, blood diseases, respiratory diseases, neurological or sensory diseases, digestive diseases, genitourinary disease, cancer, injuries, or other diseases. At the end of the check-up, the doctor asked the worker to sign the declaration that they had not withheld elements relevant to their health at work.

Furthermore, workers were invited to participate in a mental health promotion activity, which involved completing a questionnaire on the perception of anxiety, depression, work stress, and work ability. While the medical examination with a judgement of suitability is mandatory to continue working activity, participation in the health promotion projects that the occupational doctor offers is free. Workers were asked to fill out a questionnaire; based on that, the doctor offered some advice, which could include performing diagnostic tests or treatments at the facilities of the National Health Service, which in Italy is universal and free. No incentives nor disincentives were associated with participation, which was not solicited. The data provided by workers were confidential and could be communicated to company management or workers' representatives only in a collective anonymous form. Decades of experience with this health promotion scheme indicate that worker participation in health promotion activities is very high (on average over 85%) [71]. In this specific case, the participation rate was 90.1%. The reasons given by workers for non-participation were mainly the lack of time to fill out the questionnaire. Only two workers justified their refusal to participate with skepticism toward the improvement of working conditions. In this study, we only considered workers who participated in both the mandatory medical examination and the voluntary completion of questionnaires.

This study was conceived as a census of all workers subjected to health surveillance. For ethical reasons, in fact, the opportunity to participate in health promotion must be offered to the entire population. Consequently, we did not calculate the sample size. Informed consent was obtained from all subjects involved in the study.

### 2.2. Questionnaire

Anxiety and depression symptoms were measured with the Italian version [72] of the Goldberg Anxiety and Depression Questionnaire (GADS) [73]. The questionnaire includes a series of 9 binary response questions for each of the two sub-scales. This questionnaire was designed by Goldberg as a tool through which the general practitioner could obtain some indication of the presence of anxiety or depression in their patients. Each of the two Anxiety and Depression subscales can be considered a continuous variable, with values ranging from 0 to 9; higher values indicate greater levels of anxiety or depression. Moreover, the author of the questionnaire stated that “A patient with a score at the cutoff for either scale (that is, five symptoms of anxiety or two symptoms of depression) has a 50% chance of having a clinically important disturbance, and above these scores, the probability rises sharply” [73]. In line with this statement, we considered that there was an over 50% probability that a subject with 6 points or more on the anxiety scale was anxious, and one scoring 3 points or more on the depression scale was depressed. In this way, we constructed the binary categories “anxious/not anxious” and “depressed/not depressed”. The reliability of the GADS questionnaire in our survey, calculated with Cronbach's alpha, was 0.843 for anxiety and 0.803 for depression.

Work stress was measured with the Italian version [74],[75] of the Siegrist Effort/Reward Imbalance (ERI) questionnaire [76],[77]. The questionnaire consists of four-point Likert-type questions. The effort made to work (Effort) is the result of three items, with scores ranging from 3 to 12. Seven questions measure the material or immaterial rewards derived from work (Reward) (score 7–28). Occupational or extrinsic stress is measured as a weighted rate between Effort and Reward (ERI). Furthermore, the Overcommitment scale, which expresses intrinsic stress or excessive commitment to work [78],[79], is composed of 6 questions (score range 6–24). Cronbach's alpha in this investigation was 0.782 for Effort, 0.729 for Reward, and 0.774 for Overcommitment.

Satisfaction with work was measured by a single question (range 1–7) taken from the Italian version [80] of the questionnaire by Warr et al. [81].

Work ability was measured with the Italian version [82] of the Work Ability Index (WAI) [83]. The WAI has 10 items that comprise 7 components and include questions such as “Assuming that your work ability at its best has a value of 10 points, how many points would you give your current work ability?”. The WAI score ranges from 7 to 49 and, according to the authors' indications [83],[84], can be classified as excellent (WAI 44–49), good (WAI 37–43), moderate (WAI 28–36), and poor work ability (WAI ≤ 27). Reliability measured by Cronbach's alpha in this investigation was 0.653, i.e., just acceptable. In the various studies conducted with this tool, it ranged from 0.54 to 0.83 [85]–[90].

### 2.3. Statistics

The distribution of the variables of interest was tested for normality using the Kolmogorov–Smirnov and Shapiro–Wilk tests. The internal consistency of the questionnaires was evaluated using Cronbach's alpha.

The comparison between the group means was made with an analysis of variance (*ANOVA*). In the case of a positive *ANOVA* test, post-hoc comparisons were made with the Bonferroni test. The comparison of categorical data was performed using Pearson's chi-square test.

To evaluate to what extent anxiety or depression scores, or anxious or depressed status, determined the risk of being classified as a subject with poor work ability, binary regression models were constructed in which the dependent variable was poor work ability status. First, univariate models adjusted for age and sex were tested, in which each predictor (anxiety, depression, anxious, depressed) was entered one at a time to determine the odds ratio (*OR*) and the 95% confidence interval (95% *CI*). Then, two multivariable hierarchical logistic regression models were built; in the first, age, sex, anxiety, and depression were predictors; in the second, extrinsic (ERI) and intrinsic stress (Overcommitment) were added.

### 2.4. Ethics approval of research

The study was conducted in compliance with the Declaration of Helsinki and the principles of the ICOH Code of Ethics for Prevention Operators. The project was approved by the Ethics Committee of the Catholic University of the Sacred Heart (n. 2896 of 5 December 2019).

## 3. Results

In this work, 1064 HCWs from a public health company underwent their annual medical examination and were invited to join the health promotion project; 959 participated in the survey (90.1%). The mean age was 44.59 ± 12.74 years; 639 were female workers (66.6%). Overall, 80 workers reported they were being treated for MD (Group A, 8.3% of the sample) and 55 said they suffered from MD but were not being treated (Group B, 5.7%). These workers were compared with 824 colleagues who did not report mental disorders during the medical examination in the workplace (Group C, control group).

Table 1 reports the characteristics of the three groups and the scores of the variables examined. Workers who reported MD had an average age significantly higher than the control group (*p* = 0.011). There were no gender differences between the groups. Perceived occupational stress was significantly higher in workers with MD than in the control group (*p* < 0.001). Compared to controls, workers with MD reported greater efforts to comply with work requests (Effort, *p* < 0.001) and fewer rewards received for work performed (Reward, *p* < 0.001). Consequently, the weighted rate between effort and reward, i.e., work stress (ERI), was significantly greater in workers with MD than in others (*p* < 0.001). Also, intrinsic stress (Overcommitment) was significantly higher in employees affected by mental health problems than in controls (*p* < 0.001). Levels of anxiety and depression were higher among workers who reported having MD than in their colleagues (*p* < 0.001). Job satisfaction was significantly lower in MD workers than in controls (*p* < 0.001). Work ability was significantly worse in MD workers than in controls (*p* < 0.001). Workers with untreated MD had worse values of stress, overcommitment, anxiety, depression, job satisfaction, and work ability than those who were in treatment, even if the difference was not significant.

Using the GADS questionnaire, we investigated the distribution of anxiety and depression symptoms in the sample under examination. According to the aforementioned cutoff criterion, there were 328 (34.2%) suspected cases of anxiety and 334 (34.8%) of depression. As expected, groups A and B had a much higher prevalence of cases of anxiety or depression than controls. Three out of four of those who reported MD suffered from anxiety and/or depression. Even among workers who did not report MD, there were many cases that could have been diagnosed as anxious, depressed, or both (Table 2). Workers with a suspected anxiety disorder (*n* = 316 cases) had significantly lower work ability than other workers (36.09 ± 5.78 in anxious workers *vs*. 39.62 ± 4.99 in other workers, *p* < 0.001). Similarly, depressed employees (*n* = 323) had a significantly lower WAI score than healthy workers (36.62 ± 5.73 *vs*. 39.55 ± 5.08, *p* < 0.001).

**Table 1.** Comparison between workers with and without mental health problems.

**Project**	**Group A (treated for MD)**	**Group B (reporting MD)**	**Group C (control)**	***p***
**Age**	46.8 ± 11.0	48.6 ± 11.9	44.1 ± 12.9	0.011* A *vs*. C = 0.033 B *vs*. C = 0.033
**Gender (M)** **(F)**	22 (6.9%) 58 (9.1%)	15 (4.7%) 40 (6.3%)	283 (88.4%) 541 (84.7%)	0.285^#^
**Effort**	8.82 ± 2.24	9.17 ± 2.40	7.98 ± 2.17	<0.001* A *vs*. C = 0.003 B *vs*. C < 0.001
**Reward**	16.68 ± 3.31	15.72 ± 3.56	18.19 ± 3.38	<0.001* A *vs*. C < 0.001 B *vs*. C < 0.001
**Stress (ERI)**	1.31 ± 0.49	1.48 ± 0.64	1.08 ± 0.44	<0.001* A *vs*. C < 0.001 B *vs*. C < 0.001
**Overcommitment**	16.35 ± 3.59	16.96 ± 3.76	13.41 ± 3.46	<0.001* A *vs*. C < 0.001 B *vs*. C < 0.001
**Anxiety (GADS)**	5.98 ± 2.95	6.64 ± 2.29	3.60 ± 2.79	<0.001* A *vs*. C = 0.003 B *vs*. C < 0.001
**Depression (GADS)**	4.29 ± 2.62	5.00 ± 2.37	2.35 ± 2.27	<0.001* A *vs*. C < 0.001 B *vs*. C < 0.001
**Job satisfaction**	3.83 ± 1.43	3.75 ± 1.66	4.50 ± 1.52	<0.001* A *vs*. C = 0.001 B *vs*. C = 0.002
**Work ability (WAI)**	32.49 ± 5.55	33.43 ± 6.56	39.35 ± 4.88	<0.001* A *vs*. C < 0.001 B *vs*. C < 0.001

Note: * ANOVA with Bonferroni post-hoc comparison. ^#^ Chi-square test.

**Table 2.** Prevalence of cases of anxiety or depression disorder.

**Project**	**Group A (treated for MD)**	**Group B (reporting MD)**	**Group C (control)**	***p***
**Anxious**	50 (62.5%)	36 (65.5%)	242 (29.4%)	<0.001^#^
**Depressed**	51 (63.7%)	40 (72.7%)	243 (29.5%)	<0.001^#^

Note: ^#^ Pearson's chi-square test.

Workers with poor work ability were more numerous in the control group than in the groups of subjects who declared mental problems; however, the prevalence of poor work ability was significantly higher among those with MD than among other workers (*p* < 0.001). Also in this case, the highest share of employees with poor work ability was found among those who were not in treatment (Table 3).

**Table 3.** Prevalence of workers with poor work ability

**Project**	**Group A (treated for MD)**	**Group B (reporting MD)**	**Group C (control)**	***p***
**Poor work ability**	12 (15.0%)	11 (20.8%)	16 (2.0%)	<0.001^#^
**Moderate, good, excellent work ability**	68 (85.0%)	42 (79.2%)	785 (98.0%)	

Note: ^#^ Pearson's chi-square test.

To analyze the impact of anxiety and depression on poor work ability, we used hierarchical logistic regression analysis with poor work ability as the binary dependent variable. Initially, we built univariate models, entering anxiety and depression as predictors separately and adjusting for age and gender (Table 4). Both anxiety and depression scores were found to be highly significant predictors of poor work ability (*p* < 0.001). In Table 4, we also calculated the risk of a poor WAI associated with a diagnosis of suspect anxiety or depression; those identified as anxious at screening had 8 times greater risk of severe disability (*p* < 0.001), and those identified as depressed had 4.5 times greater risk of poor work ability than controls (*p* < 0.001).

**Table 4.** Association of anxiety and depression with poor work ability. Univariate logistic regression models adjusted for age and gender.

**Project**	***Odds ratio***	**95% *confidence interval***
**Anxiety**	1.54***	1.33–1.78
**Depression**	1.49***	1.31–1.71
**Anxious worker**	8.11***	3.74–17.58
**Depressed worker**	4.49***	2.22–9.10

Note: ***p < 0.001; **p < 0.01; *p < 0.05.

By means of multivariate hierarchical logistic regression analysis, we then studied the risk of being in a condition that requires the re-establishment of work ability. We set poor work ability status as the dependent variable. First, we inserted the demographic variables and subsequently anxiety and depression as independent variables (Model I); then, we added occupational extrinsic and intrinsic stress factors (Model II). Model I indicated that a status of poor work ability could be determined by age (*OR* = 1.08; 95% *CI* = 1.04–1.12, *p* < 0.001) and by anxiety (*OR* = 1.38; 95% *CI* = 1.13–1.68, *p* < 0.001). The presence of symptoms of depression was associated with a non-significant increase in the risk of having poor work ability. In Model II, age and anxiety maintained their significant role as predictors of poor work ability, and overcommitment was added as a significant independent predictor of poor work ability (*OR* = 1.20; 95% *CI* = 1.07–1.35, *p* < 0.001) (Table 5).

**Table 5.** Factors associated with poor workability. Multivariate hierarchical logistic regression.

**Project**	**Model I *odds ratio***	**95% *CI***	**Model II *odds ratio***	**95% *CI***
**Age**	1.08***	(1.04–1.12)	1.08***	(1.04–1.13)
**Gender**	0.86	(0.42–1.79)	0.95	(0.44–2.05)
**Anxiety**	1.38***	(1.13–1.68)	1.31*	(1.06–1.62)
**Depression**	1.17	(0.96–1.42)	1.08	(0.88–1.33)
**ERI**			0.57	(0.26–1.26)
**Overcommitment**			1.20***	(1.07–1.35)
***R^2^***		0.242		0.274

Note: ***p < 0.001; **p < 0.01; *p < 0.05.

As evidenced in Table 1, workers who reported being treated for mental disorders at their periodic medical examination in the workplace had significantly lower levels of work ability than those of their colleagues who declared good mental health (*p* < 0.001). They also reported greater work stress (high effort, low rewards, high overcommitment) and lower job satisfaction than their healthy colleagues (*p* < 0.001).

Workers in treatment had no significant differences in GADS anxiety and depression scores compared to colleagues with symptoms but not in treatment. These symptomatic but untreated workers reported significantly lower work capacity (*p* < 0.001) and greater occupational stress (*p* < 0.001) than their healthy colleagues.

The presence of symptoms of anxiety and depression in HCWs is associated with low work ability, high stress, and low job satisfaction both in diagnosed and treated workers and in those who were not in treatment. Health surveillance in the workplace should screen for psychiatric disorders in HCWs so as to assist in maintaining high levels of care.

## 4. Discussion

Our study revealed a high prevalence of clinical or subclinical anxiety and depression in HCWs. Screening with the GADS questionnaire indicated that, in our sample, 34.2% of operators were affected by subclinical or clinical anxiety and 34.8% by depression. These prevalence rates, obtained in a public health company before the COVID-19 pandemic, fall within the range of values estimated by meta-analytic studies conducted during the COVID-19 pandemic when the mental health problem of HCWs was clearly visible to all (Table 6). An umbrella review of 72 meta-analyses conducted among HCWs during the pandemic revealed that the pooled prevalence of anxiety was 31.8% (95% *CI* 29.2–34.61), and 29.4% (95% *CI* 27.13–31.84) for depression [91]. A systematic review and meta-analysis summarizing these studies highlighted the effect that this state of poor mental health can have on the functioning of healthcare and identified feelings of depression and anxiety among the factors associated with poor work performance [92].

**Table 6.** Pooled prevalence (and 95% confidence interval) of mental disorders in health care workers during the COVID-19 pandemic.

**Authors**	**Anxiety Prevalence (95% *CI*)**	**Depression Prevalence (95% *CI*)**	**PTSD Prevalence (95% *CI*)**	**Insomnia Prevalence (95% *CI*)**
**Salari N et al. 2020 [18]**	25.8% (20.5–31.9)	25.8% (20.5–31.9)	n.r.	n.r.
**Pappa S et al. 2020 [1]**	23.2%	22.8%	n.r.	n.r.
**Serrano-Ripoll MJ et al. 2020 [22]**	30% (30–31)	24% (24–25)	13% (13–14)	n.r.
**Li Y et al. 2021 [3]**	22.1% (18.2–26.3)	21.7% (18.0–25.2)	21.5% (10.5–34.9)	n.r.
**Marvaldi M et al. 2021 [2]**	30.0% (24.2–37.05)	31.1% (25.7–36.8)	20.2% (9.9–33.0)	44.0% (24.6–64.5)
**Saragih ID et al. 2021 [12]**	40% (29–52)	37% (29–45)	49% (22–75)	n.r.
**Wu T et al. 2021 [17]**	31.4%	31.9%	n.r.	37.9%
**Norhayati MN et al. 2021 [23]**	34.81% (30.80–38.83)	34.61% (30.87–38.36)	15.29% (11.43–19.15)	37.89% (25.43–50.35)
**Arora T et al. 2022 [24]**	28% (21–36)	22% (13–33)	33% (0–86)	n.r.
**Chen Y et al. 2022 [16]**	43% (36–50)	45% (37–52)	n.r.	n.r.
**Lee BEC et al. 2023 [25]**	28.7% (26.5–31.0)	28.5% (26.3–30.7)	25.5% (22.5–28.5)	24.4% (19.4–29.9)
**Ghahramani S et al. 2023 [26]**	47% (22–74)	36% (24–50)	37% (19–59)	49% (28–70)

Note: n.r.=not reported

Not without foundation, comments on the numerous studies conducted during the pandemic have linked anxiety and depression of HCWs to fear of infection [93] or to the varying occupational risks associated with the waves of the pandemic [94]–[98]. These psycho-pathogenic factors were certainly absent in our observations, collected before the outbreak of the pandemic. We must therefore compare our results with those obtained before the pandemic. Unfortunately, before 2020, studies on anxiety and depression among HCWs were rare, and meta-analytic evaluations were mostly sectoral. For example, Petrie et al. [99] estimated prevalence rates of 15% for depression, 15% for anxiety, and 27% for general psychological distress among ambulance personnel. A high pooled prevalence of depression (34.0%) was reported among nursing students, with an even higher prevalence (41.0%) in younger students [100]. High pooled rates of depression (30.6%) and anxiety (32.9%) were estimated in Brazilian medical students [101]. The published studies focused mainly on nurses, who showed the highest relative risk of depression (*RR* = 3.5, 95% *CI* = 1.3–9.6) compared to other human service professions and other healthcare workers [102]. The prevalence of anxiety and depression was reported to be 41.2% and 32.4% among Australian nurses [103], 28.7% and 16.6% among emergency nurses in Oman [104], and 27% and 19.6% among psychiatric nurses in Ghana [105]. A Chinese study showed that physicians had a higher risk of being treated for anxiety, depression, and sleep disorders than the general population [106]. A summary of the literature indicated that even before the pandemic, many HCWs were showing burnout, with the highest prevalence rates among nurses, younger persons, and trainees [107]. The paucity and heterogeneity of data make it impossible to understand to what extent differences in study designs, sample sizes, healthcare settings, cultural factors, or the measurement tools used for anxiety and depression have influenced the prevalence values recorded. It should be noted that the rates we recorded did not refer to a psychiatric diagnosis but to the risk of being classified as suffering from anxiety and depression by a psychiatrist. Overall, our findings highlight that subclinical or clinical mental health problems were largely prevalent among HCWs before the pandemic. There is no reason to believe that the problems observed will not persist after the pandemic has subsided.

It is important to remember that studying the mental health of HCWs is not easy due to the stigma of mental illness and the impact that these illnesses have on work ability. To the best of our knowledge, we failed to find any studies that associated these non-negligible rates of anxiety and depression with work ability.

Although HCWs can be exposed to many factors that increase the risk of developing MD and are also known to suffer from a high prevalence of anxiety or depression, it is worth pointing out that few studies have attempted to ascertain whether the mental health of HCWs is associated with work ability. In the literature, the relationship between anxiety/depression and work ability has been investigated mainly in groups of patients who returned to work after treatment or who were followed for a certain period during their psychotherapy. In a prospective study on 83 women returning to work after breast cancer, the WAI was negatively associated with symptoms of depression and anxiety [108]. An Italian study of women with breast cancer returning to work found that baseline levels of anxiety and depression were significantly and inversely associated with work ability [109]. Anxiety was negatively associated with the WAI among adult patients who returned to work after systemic autoimmune myopathies [110]. In a group of Finnish patients with psychiatric disorders that was followed for 3 years after the start of treatment, work ability measured by the WAI showed a significant improvement concomitant with the improvement in their psychiatric condition [111]. In another group of German psychiatric patients, 2-year group psychotherapy led to a significant reduction in symptoms of anxiety and depression and a simultaneous improvement in the WAI score [112].

Studies conducted in the workplace are very rare and give limited information. In a cross-sectional study on 413 bank workers, psychological distress measured by the General Health Questionnaire was inversely correlated with the WAI [113]. In a sample of 57 therapeutic prison officers, psychological distress was significantly associated with poor work ability; furthermore, impaired work performance due to mental demands was significantly associated with anxiety and sleep loss [114]. The few studies that have considered HCWs have focused on their clinical status as patients. For example, in Germany, HCWs with an occupationally acquired chronic hepatitis B/C infection who participated in an inpatient rehabilitation program showed noteworthy anxiety and depression symptoms during the study period and poor work ability. This improved significantly toward the end of treatment and remained at a moderate level six months later [115]. However, it has been known for some time that mental health complaints such as stress, mild depression, and anxiety disorders, often referred to as common mental disorders, can lead to impairments in work performance [116]–[118]. These disabilities may lead not only to decreased output but can also have major repercussions in some professions such as nursing and medical care. The impaired work functioning of staff can have a negative impact on both the caregivers and their patients' health. Medication errors, needle stick injuries, near errors, and a decline in patient satisfaction are a few examples of these negative consequences [119]. Since this is a very sensitive topic, it may have restricted the publication of studies demonstrating the association of anxiety and depression with reduced work ability in HCWs and the negative consequences for patients. However, the limited number of published studies has not prevented healthcare managers from investing in programs to improve the mental health of HCWs with MDs. Some of these programs have been conducted together with occupational medicine initiatives and have proven to be more efficient and more cost-effective than those traditionally linked to psychological support alone [120]. A Dutch study demonstrated that screening HCWs for anxiety, depression, and burnout can lead to a 5–11-fold return on investment (ROI) by reducing absenteeism and presenteeism [121].

Our study demonstrated that workers treated for MD reported significantly lower work ability than their colleagues. Workers not being treated for MD had a significantly more severe degree of disability than controls; this value was also worse than in workers being treated, although the difference was not significant. Anxiety and depression were the most frequent disorders in workers reporting MD. Subclinical cases of anxiety and depression that emerged during screening had a lower level of work ability than other workers. The difference observed was particularly significant from a clinical point of view because the control group was made up not only of healthy subjects but also of workers with chronic pathologies other than MD. Therefore, anxiety and depression were the most important health factors associated with poor work ability in HCWs. Preventing these common mental disorders could lead to significant benefits for work ability and patient care.

During our study, we observed that not all workers who tested positive at screening had reported suffering from MD during their medical examination. In fact, when asked if they had MD, only 14% gave an affirmative answer; of these, just over half were under treatment. It is important to remember that this survey was conducted in a public company, among workers employed on permanent contracts regulated by a national collective labor agreement. In this population, the risk of being fired due to inability to work is very remote. This indicates that none of the interviewees had an interest in hiding the illnesses they were suffering from, but it does not exclude the possibility that some participants might have exaggerated their degree of disability in the hope of obtaining greater benefits in a system in which dismissal is a very rare event. The underreporting of anxiety and depression does not stem from a desire to hide a mental health problem, as demonstrated by the fact that workers were willing to comply with the screening test; it demonstrates instead that they considered that the disorder was not important enough to interfere with their work and therefore did not need to be reported to the occupational doctor. Serious underreporting, and especially the lack of MD treatment, are particularly disturbing in a healthcare population that should be aware that mental health problems can interfere with many fundamental aspects of healthcare practice.

Our study confirmed all our initial hypotheses, since it demonstrated that (1) employees reporting MD have worse work ability than others; (2) anxiety and depression represent the largest share of reported MD; (3) subclinical anxiety and depression are widespread among employees who underreport and undertreat these disorders; (4) subclinical anxiety and depression are associated with occupational stress, poor job satisfaction, and (5) worse work ability. In the absence of longitudinal studies that can demonstrate the direction of the relationship between MD and work ability, we can state that anxiety and depression may cause a reduction in work ability and not the opposite. The concept of work ability that we illustrated previously postulates that illnesses reduce the ability to perform the job, and not vice versa. In addition to this objective component, it must be remembered that the WAI questionnaire introduces a subjective factor since it is self-compiled. Anxiety probably determines an unfavorable evaluation of one's state of health in relation to the demands of work, while depression negatively influences one's self-evaluation of the ability to cope with the physical and psychological demands of work; overall, both contribute to determining a low value of the WAI score. Stress also intervenes in this self-evaluation. Excessive work commitment (overcommitment) contributes to depression when one's ability to cope with the demands of the job is assessed as insufficient. In fact, we have seen in this study that particularly anxious workers with excessive overcommitment often describe themselves as subjects with poor work ability who need help. Excessive commitment causes the worker to set very high and challenging goals and performances, while anxiety makes them fear not being able to achieve them. The self-assessment of poor work ability arises from a combination of these tensions.

Apart from these hypotheses, which could be the subject of future studies, our observation sheds light on the relationship between anxiety, depression, and poor work ability, on the widespread diffusion of these disorders in HCWs, and on the possibility that this may cause a worsening of the quality of care. To ensure that patients receive prompt care, the work performance and mental health of HCWs are increasingly in need of attention.

Another line of development of studies conducted in health surveillance could be to analyze the differences in stress levels, work ability, and mental health among the different categories of HCWs. A better understanding of the relationships between work organization, stress, anxiety, and depression could provide useful elements to design work ability promotion actions differentiated for the various professional categories. The main limitation of this study was its cross-sectional design that prevented us from stating with certainty that it was MD, anxiety, and depression that reduced the WAI score. The inverse relationship, that it was poor work ability that induced anxiety, depression, and other mental health problems, appears unlikely, even if confirmation can only come from longitudinal observations. Our research was conducted in only one public health company, and this limited the extension of our results to other situations. However, there is no reason to believe that workers from this company were different from other HCWs.

A weakness of this study, as with all those conducted in the workplace during health surveillance, was the limited time of action. Health surveillance must respect the time limits dictated by the production activity. For this reason, it is necessary to choose very simple and short tools, which allow for rapid compilation and immediate correction. The GADS questionnaire was designed by Goldberg for the practice of the general practitioner, who also has very tight time limits, and is ideal for work environments. The diagnostic power of the questionnaire and the optimal cutoff values were established by the author of the instrument and confirmed by many subsequent studies. It is clear, however, that the results collected with this epidemiological tool, as with other similar ones, are not equivalent to the clinical diagnoses obtained from specialist visits.

Another weakness of the study was the modest reliability of the questionnaire used to measure work ability. The WAI presents some critical issues that, despite the widespread use of the instrument, are not universally known. From a comparison of the WAI results with health surveillance data [122], it emerged that the concept of work ability is not chronologically homogeneous: some workers refer to the ability to work during the year, others to the day on which the questionnaire was filled out. Even the classification of the type of work varies unpredictably. Workers who do the same job describe it in some cases as physically demanding, in other cases as psychologically demanding, and in others as both physically and psychologically demanding [122]. Moreover, in the comparison between the current work ability compared with lifetime best younger workers, students and interns refer not to the ability possessed in the past, but to that which they hope to obtain in the future [122]. Furthermore, many workers indicate a greater number of disorders than those declared in the routine health checks they undergo to establish fitness for work [122]. Finally, a paradoxical aspect concerns gender: the WAI score is inversely proportional to sick leave and is on average lower in women than in men. However, females and males had the same number of sick leave in the previous three years [122]. This demonstrates that gender-related cultural and social factors influence self-evaluation. In another study, we demonstrated that various occupational and emotional factors may influence the WAI score [123]. All these factors contribute to reducing the reliability of the instrument, and this has been known for some time. In 2005, the large Nurses Early Exit Study, which embraced over 38,000 nurses from 10 European countries (Belgium, Germany, Finland, France, Italy, Norway, Netherlands, Great Britain, Poland, and Slovakia), obtained a Cronbach's alpha for total sample equal to 0.72, while coefficients for national samples were ranging from 0.54 for Slovakia to 0.79 for Finland [85]. Subsequent studies have not obtained better values, with Cronbach's alpha of 0.66 [124], 0.71 [125], and 0.74 [126]. Commenting on similar results to those we obtained, Kaewboonchoo and Ratanasiripong [124] observed that workers' poor knowledge of the concept of work ability tends to reduce the reliability of the questionnaire. A solution to these measurement problems could be an improved version of the WAI or the development of another, more reliable measuring tool.

## 5. Conclusions

The state of widespread mental distress among HCWs is a problem of particular importance, not only for the well-being of workers but also for the care of patients. Workplace health surveillance services should screen workers for anxiety and depression and plan mental health promotion interventions. Employers have a vested interest and economic advantage in supporting these mental health promotion programs in HCWs.

## Use of AI tools declaration

The authors declare they have not used Artificial Intelligence (AI) tools in the creation of this article.
